# Well, How Did We Get Here? The Current State of Pediatric Endocrinology

**DOI:** 10.1016/j.jpedcp.2026.200238

**Published:** 2026-08-17

**Authors:** Perrin C. White

**Affiliations:** Division of Pediatric Endocrinology, UT Southwestern Medical Center, Dallas, TX

**Keywords:** workforce, physician shortage, fellowship training, health care reimbursement, value-based compensation

## Abstract

**Objective:**

To describe factors leading to the development of current workforce challenges in pediatric endocrinology and to propose solutions.

**Study design:**

The author made use of 32 years of experience as chief of pediatric endocrinology at a large academic medical center, review of fellowship match and workforce data from the American Board of Pediatrics, and review of compensation data from the Association of American Medical Colleges.

**Results:**

Although many find pediatric endocrinology a fulfilling career, workforce shortages lead to increased workloads, and relatively low remuneration and excessively long training requirements discourage entry into the field.

**Conclusions:**

Potential solutions include value-based remuneration supported by robust funds-flow agreements with hospitals, streamlined training requirements, and utilization of informatics to support better targeted referrals and effective comanagement of selected patients with primary care physicians.

“And you may ask yourself, ‘Well, how did I get here?’”

Talking Heads, Once in a Lifetime.^1^

Over the last several decades, pediatric endocrinology has faced progressively severe workforce challenges, in marked contrast to earlier projections that the pediatric endocrinology workforce would be in surplus in the near future.^2^ This article reviews some of the factors underlying the current situation and outlines some possible solutions.

## Historical Background

The Association for the Study of Internal Secretions was established in 1918 in the US and renamed the Endocrine Society in 1952.^3^ Pediatric endocrinology began as a subspecialty in the 1940s with the establishment of pediatric endocrinology clinics at Johns Hopkins by Lawson Wilkins and at the Massachusetts General Hospital by Nathan Talbot. Diabetes as a clinical pediatric discipline evolved in parallel, starting in clinics at Harvard, the University of Iowa, Cincinnati Children's Hospital, and St. Louis Children's Hospital. In the US, the Pediatric Endocrine Society, initially named the Lawson Wilkins Pediatric Endocrine Society, was formed in 1972. European and Japanese societies were formed within the same time period.^3^^,^^4^ Endocrinology was established as a subspecialty by the American Board of Pediatrics (ABP), with its first certification examination in 1978; today, there are more than 1000 board-certified pediatric endocrinologists in the US.

## Current State of the Workforce

If not otherwise stated, data are extracted from the ABP database, which surveys diplomates at the time of recertification.^5^

### Geographic Distribution

**Figure 1:**
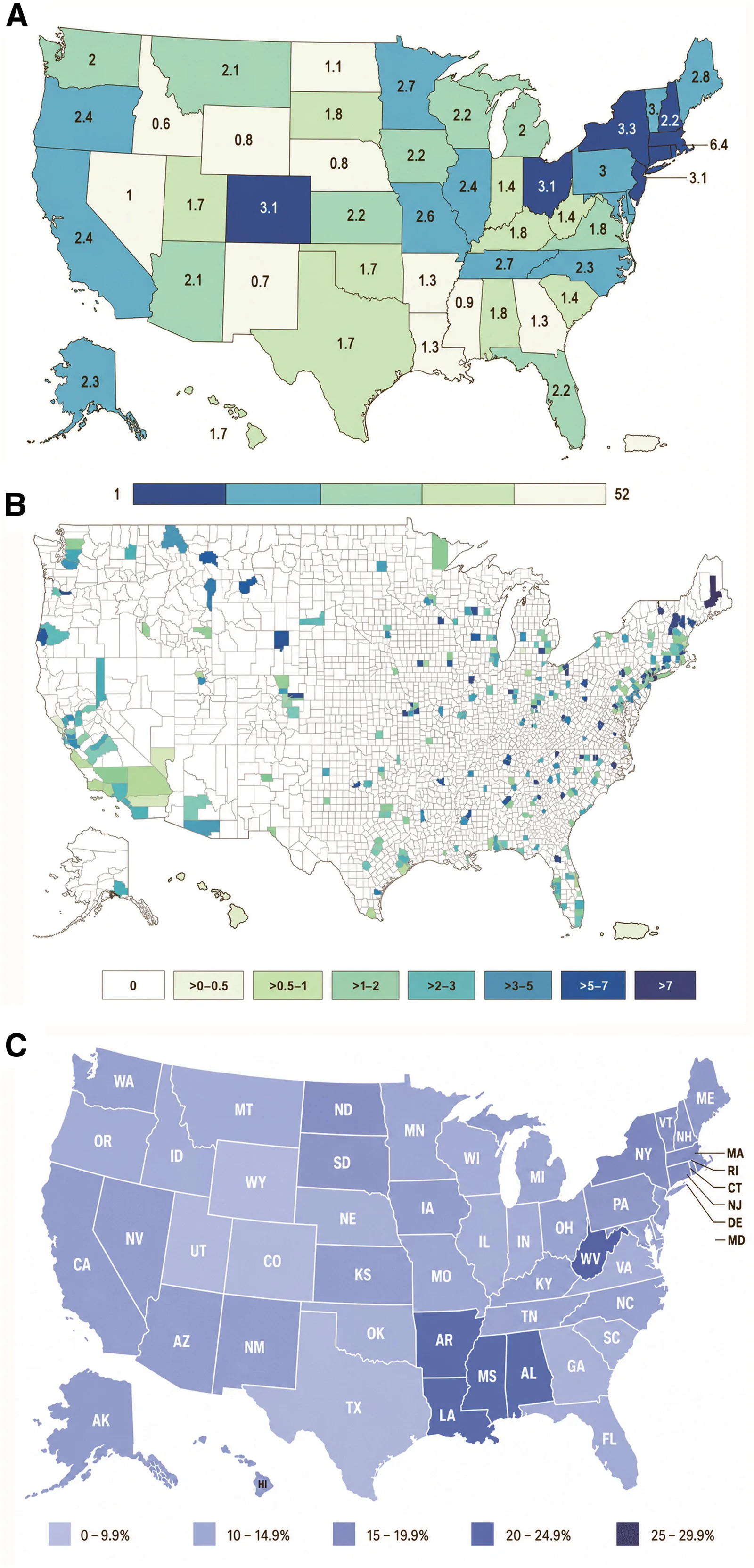
**A,** Distribution of pediatric endocrinologists across the US; the numbers represent pediatric endocrinologists per 100 000 children in each state. The *color scale* reflects the relative ranking of states and territories, with *darker scale* denoting a higher ranking. **B,** Pediatric endocrinologists are concentrated in large metropolitan areas. Counties are colored according to a scale denoting the number of pediatric endocrinologists per 100 000 children. **C,** Prevalence of childhood (aged 6-17 years) obesity in 2023-2024; the *color key* denotes the percent of obese children in each state. Obesity is highest across the Southern tier states, almost all of which have relatively low numbers of pediatric endocrinologists.

There are marked disparities in the distribution of pediatric endocrinologists across the US (Figure 1, A), ranging from 0.6 per 100 000 children (aged 0-17 years) in Idaho to 10.3 in Washington DC. Moreover, pediatric endocrinologists are concentrated in large metropolitan areas (Figure 1, B). Patients residing in rural areas may need to travel long distances for an in-person visit; in 2019, the 75th and 90th percentiles for distance to the nearest pediatric endocrinologist were 28 and 81 miles, respectively.^6^

Panels **A** and **B** are adapted from the American Board of Pediatrics.^92^ Panel **C** is adapted from the Robert Wood Johnson Foundation.^93^

Disparities in demand may exacerbate limited availability. For example, several comorbidities of childhood obesity, including type 2 diabetes and dyslipidemia, are often managed by pediatric endocrinologists. However, the prevalence of childhood obesity is highest across the Southern tier states, almost all of which have relatively low numbers of pediatric endocrinologists (Figure 1, C).

### Practice Setting and Workload

**Figure 2:**
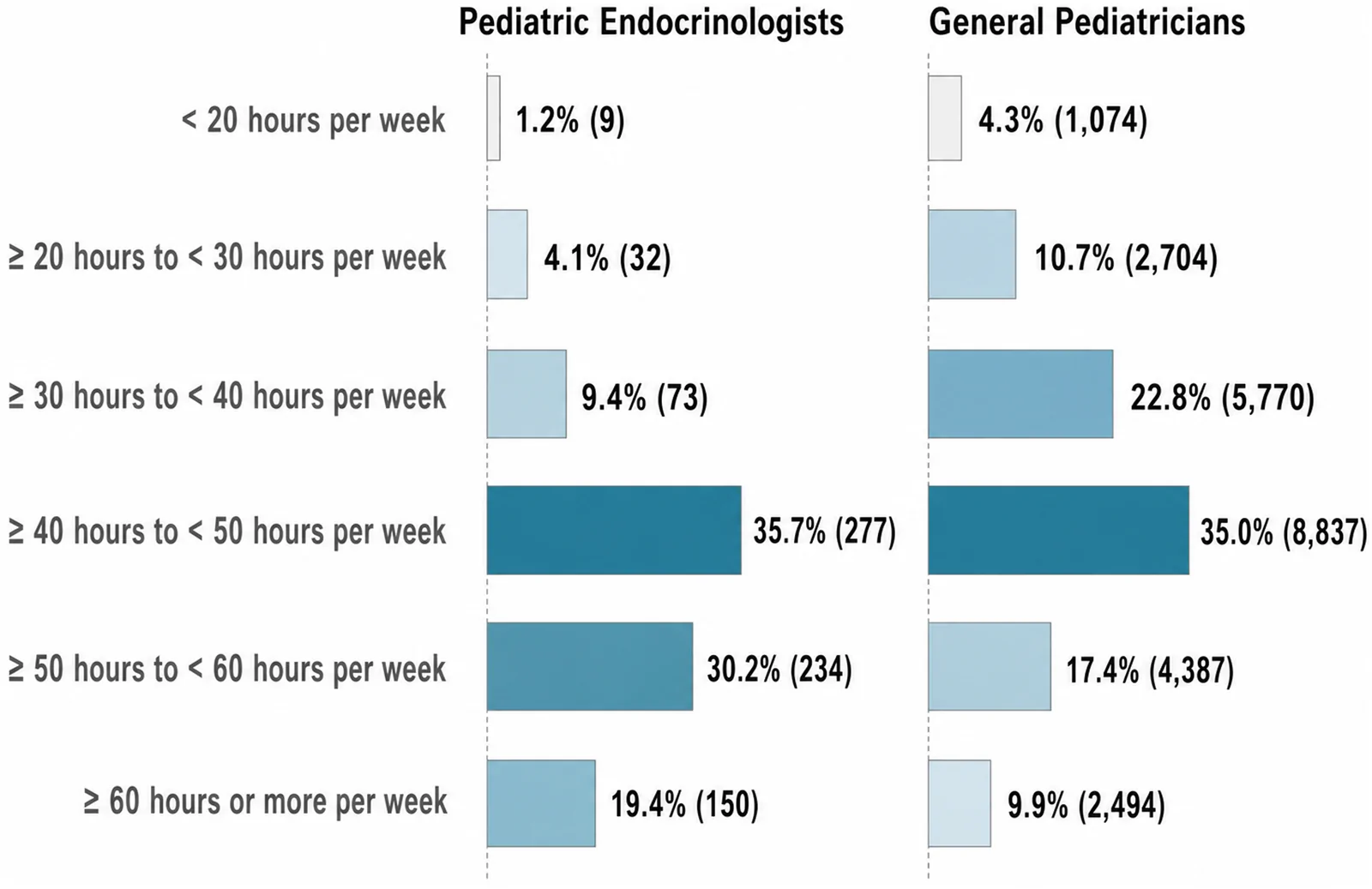
Distribution of hours worked per week for full-time pediatric endocrinologists (*left*) and general pediatricians (*right*) in 2020-2024. Percentages in each category are shown, with the absolute number of physicians in parentheses.

Adapted from the American Board of Pediatrics.^74^

**Figure 3:**
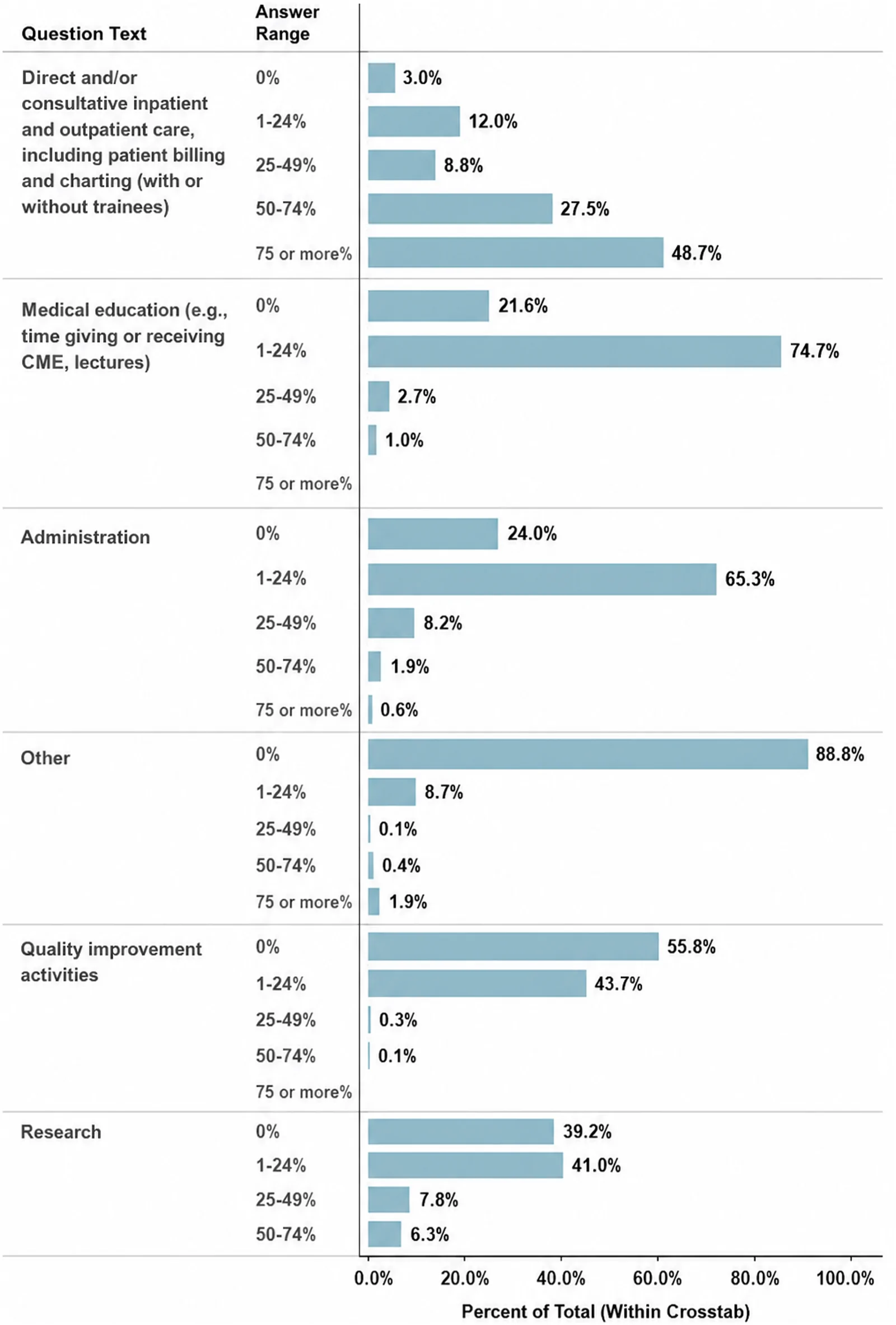
Percentages of time spent by full-time pediatric endocrinologists in various activities.

Adapted from the American Board of Pediatrics.^74^

Most—84%—pediatric endocrinologists work full time. Compared with general pediatricians, we work longer hours (Figure 2), with 56% working more than 50 hours per week, excluding home call. Most of us—76%—spend the majority of our time in direct patient care (Figure 3). Relatively, few of us spend more than 25% of our time on any other single activity: 14% on research, 4% on medical education, 11% on administration, and 0.4% on quality improvement activities. Thirty-six percent are based at universities or medical schools, with almost all of the remainder in hospital clinics or multispecialty group practices.

### Fellowship Training Trends

**Figure 4:**
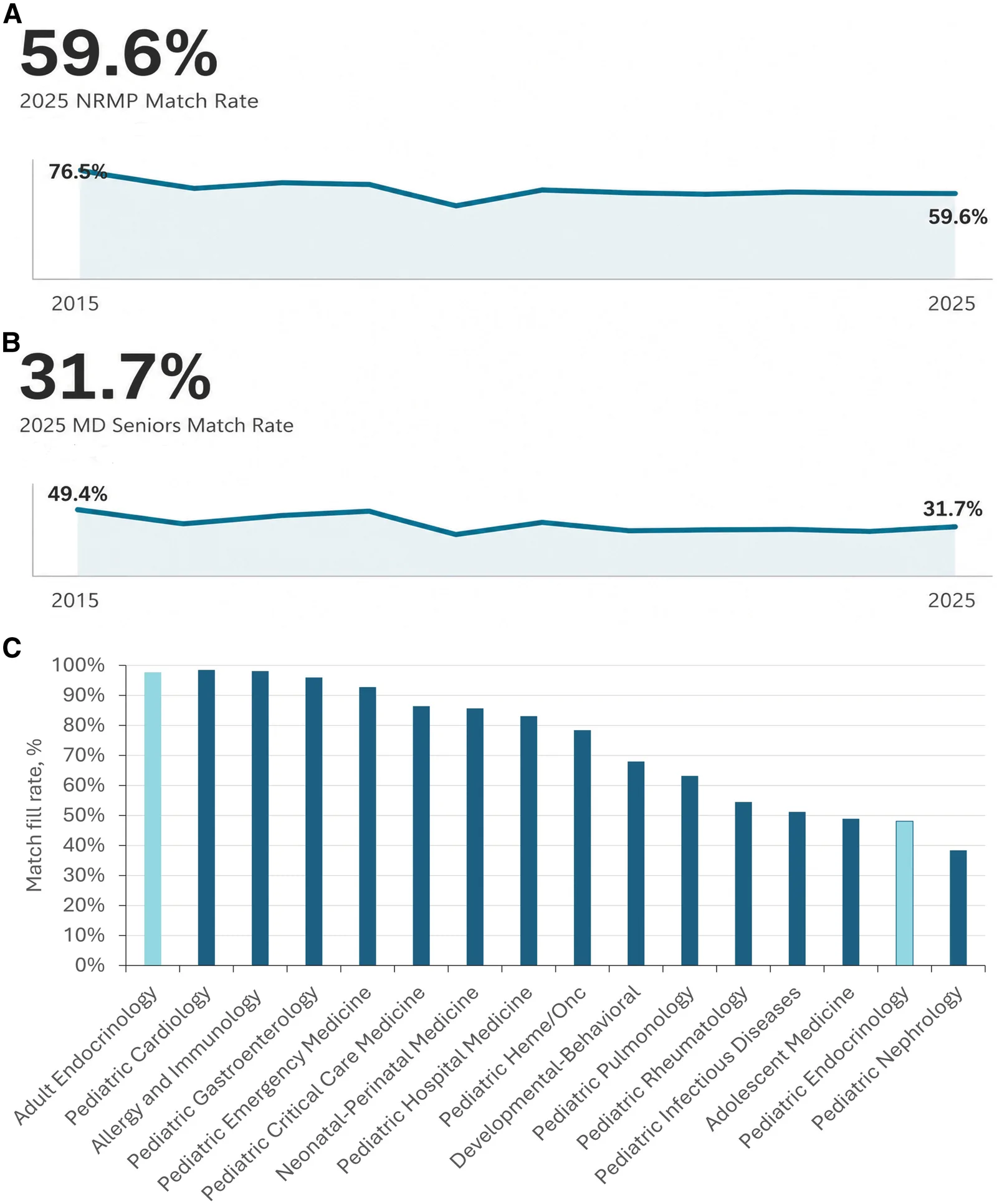
Pediatric endocrinology fellowship National Resident Matching Program trends over time. **A,** Percentage of total fellowship positions filled in the match. **B,** Percentage of total fellowship positions filled by American MD graduates. **C,** 2025 fellowship match rates for different pediatric subspecialty fellowships, plus adult endocrinology.

A review of pediatric endocrinology fellowship trends from 2015 to 2025 paints an alarming picture (Figure 4). The percentage of the ∼100 US fellowship positions filled by the National Resident Matching Program has decreased during that period from 77% to 60%. The proportion of open positions filled by American allopathic (MD) medical school graduates has declined from 49% to 32%. The rest of the matched positions are filled by osteopathic (DO) medical school graduates or by international medical graduates. Many of the latter enter training on visas that may require them to return to their home countries or serve in geographically restricted areas, thus limiting their ability to remedy staffing shortages in many locales. The number of fellowship programs in the match increased from 57 to 65, but the number of programs that fill all their positions decreased from 40 to 31. For comparison, pediatric critical care medicine fills 99% of its positions, and adult endocrinology fills 98%, albeit with a similar decrease over time in the proportion of American MD graduates entering the field.^7^

Data and graphics for **A,** and **B,** are adapted from the American Board of Pediatrics^94^; data for **C,** are adapted from the National Resident Matching Program.^95^

**Figure 5:**
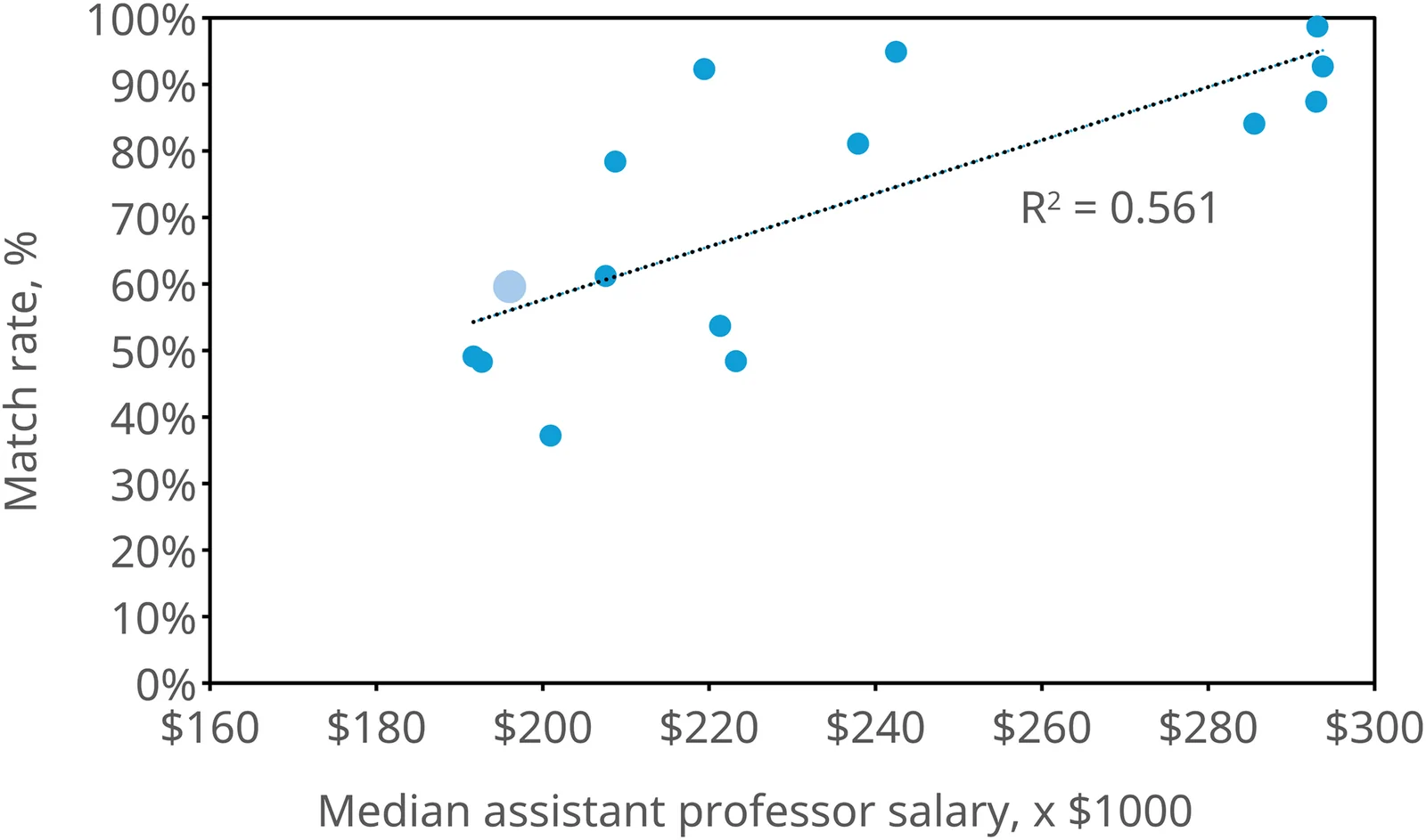
National Resident Matching Program 2025 fill rate for pediatric subspecialty fellowships (data from the American Board of Pediatrics^94^ plotted against median total compensation for an assistant professor in each subspecialty at US medical schools in 2025 [compensation data from the Association of American Medical Colleges]^10^). The *symbol* denoting pediatric endocrinology is larger and filled in a *lighter shade*. A *linear trend line* and coefficient of determination (R^2^) are shown.

Factors contributing to this situation include low remuneration relative to other specialties and pediatric subspecialties and a high clinical workload leading to burnout.^8^^,^^9^ Figure 5 plots 2025 National Resident Matching Program fill rates against 2025 median academic total compensation for assistant professors in the various pediatric subspecialties in US medical schools, collected by the Association of American Medical Colleges in their Medical School Faculty Salary Survey.^10^ The 2 quantities are highly correlated. Similarly, strong correlations exist for other metrics, such as the total proportion of fellowship positions filled (including those filled outside of the match), and for more complex measures of remuneration, such as the lifetime net present value, which represents an estimate of the financial returns that a graduating pediatric resident might expect from fellowship training followed by a career as a subspecialist.^11^ It was estimated in 2018 that the lifetime net present value of a pediatric endocrinologist was $1.5 million lower than that of a general pediatrician, one of the lowest of any pediatric subspecialty, whereas a pediatric cardiologist could expect to earn almost $900 000 more than a general pediatrician over their lifetimes. These disparities were much worse than when first studied in 2007.

We will consider these factors in detail in the following sections of this article.

### Factors Affecting Demand for Pediatric Endocrinologists

Increased demand for services exacerbates the consequences of decreased availability of pediatric endocrinologists.^12^ The explanations for this increased demand are multifactorial.

#### Increased Therapeutic Complexity

Endocrinologic diseases are an active area for drug development; 4% (22 drugs) of the 500 novel drugs approved by the US Food and Drug Administration (FDA) since 2015 are for endocrinologic indications, and roughly one-half of these are now routinely used by pediatric endocrinologists. Several are discussed in the following paragraphs. Although it is exciting to have an augmented therapeutic armamentarium, one must consider the learning curve in deploying novel therapies and the likelihood that primary care physicians will lack the expertise to use them, necessitating more subspecialty referrals. Moreover, many new therapies are expensive, provoking resistance from payors and increasing administrative burdens for prior authorizations and appeals.

#### Increasing Incidence and Prevalence of Treated Conditions

Much of the caseload for most pediatric endocrinologists consists of patients with either type 1 or type 2 diabetes.

##### Type 1 Diabetes

The incidence of type 1 diabetes in the US was 22 cases/100 000 population/year in 2018.^13^ This corresponds to a prevalence of ∼1 in 300 children younger than 18 years. The incidence has increased over time both in the US^13^, ^14^, ^15^ and worldwide.^16^, ^17^, ^18^, ^19^ The increased technological complexity of modern diabetes care makes it challenging to treat these patients in a primary care environment. The modern standard of care^20^ consists of continuous glucose monitoring, which is often available for remote monitoring by the clinician, and a hybrid closed-loop pump that may be complicated to optimize depending on the pump's algorithm and that may require frequent troubleshooting. This ideally requires a multidisciplinary care team including endocrinologists, certified diabetes care/education specialists, dieticians, and mental health professionals, the costs of which may be poorly reimbursed by many insurance programs.^21^

Moreover, advances in our understanding of the immunopathogenesis of type 1 diabetes have prompted intensive investigations of immunotherapy for both clinically apparent (stage 3) disease and for preclinical (stage 2) disease in which autoimmune attack on pancreatic beta cells is underway, but the patient is not yet markedly hyperglycemic. Teplizumab, a humanized anti-CD3 monoclonal antibody, has been approved by the FDA both to delay the progression of stage 2 diabetes to stage 3 and to prolong the honeymoon in patients aged 8-17 years with newly diagnosed stage 3 diabetes.^22^ The availability of a disease-modifying therapy for preclinical type 1 diabetes has spurred efforts to implement general population-based screening^23^, ^24^, ^25^, ^26^, ^27^ to identify at-risk individuals from the general population who might benefit from preventative interventions. Current screening methodology entails blood testing for autoantibodies against insulin and/or beta-cell proteins. A US study reported that approximately 1% of screened children have at least 1 high affinity autoantibody, and one-half of these have multiple autoantibodies^23^; it is likely that many, if not all of these, children will be referred to pediatric endocrinologists for counseling and ongoing monitoring.^28^ Although these tasks can probably be accomplished by trained nurse practitioners, this could potentially represent a significant strain on clinical resources as population screening becomes more widespread. As of mid-2026, the extent to which teplizumab therapy will be adopted by patients with newly diagnosed stage 3 diabetes, their families, and insurers is not known. A therapeutic course for stage 2 diabetes is 14 daily infusions; in contrast, the treatment for stage 3 diabetes is 12 daily infusions of the drug <8 weeks from diagnosis, repeated 6 months later, totaling 24 infusions per patient. This could potentially amount to thousands of infusions per year in a busy children's hospital, representing a strain on facilities and personnel, but also a potential source of income.

##### Obesity and Type 2 Diabetes

The prevalence of childhood obesity in the US has increased dramatically over the past 30 years.^29^^,^^30^ In 2013-2014, 9% of infants and toddlers and 17% of children and adolescents aged 2-19 years were obese, and the prevalence was more than 20% in most states in the southern US (Figure 1, C). The risk of type 2 diabetes has increased correspondingly. In the US, the adjusted incidence of type 2 diabetes among children and adolescents nearly doubled from 2002-2003 to 2017-2018^13^; it is currently 22/100 000 per year in females and 14/100 000 in males. Remarkably, the incidence of type 2 diabetes in 2017-2018 among individuals aged 15-19 years exceeded that of type 1 diabetes. The incidence and prevalence of type 2 diabetes are also increasing in Europe^31^^,^^32^ and Asia.^33^

Leaving aside consideration of the social determinants of the obesity epidemic, modern obesity treatment requires a multidisciplinary approach including nutritional counseling, exercise instruction, and—if necessary—medical management that may be beyond the capacity of many primary pediatric practices. The availability of glucagon-like peptide 1 (GLP-1) agonists and effective bariatric surgery for the management of obesity and type 2 diabetes has increased the interest of many families (and their primary care physicians) in seeking medical care for children with obesity. It is obviously beyond the capacity of pediatric endocrinologists to treat a condition occurring in 20% of children, and thus, clinical resources must be devoted to triaging referrals to identify the children with obesity-related comorbidities who could benefit most from pediatric endocrinology care.

#### Increased Scope of Practice

Several severe chronic conditions have seen marked improvements in treatment, leading to increased survival and correspondingly increased prevalence of endocrine comorbidities that were previously rare. Moreover, there are new opportunities for endocrinologists to deploy novel therapies for several chronic conditions. Such conditions may be optimally managed using multidisciplinary care models that in some circumstances may generate scheduling constraints on several different subspecialists for each patient. One example is cystic fibrosis (CF), in which the development of CF transmembrane conductance regulator modulators has increased median lifespan to more than 60 years, leading to increased numbers of cases of CF-related diabetes.^34^ Another is adrenoleukodystrophy, a genetic form of adrenal insufficiency associated with neurologic deterioration that was previously fatal, which may now be treated with allogeneic bone marrow transplantation or gene therapy, markedly improving survival.^35^^,^^36^ The availability of effective disease-modifying therapy has prompted the institution of newborn screening for this latter condition, leading to recognition that it is much more common (∼1 in 10 000 male births) than previously suspected. These factors increase the need to monitor and treat adrenal insufficiency, which can be detected much earlier and more consistently in screened cases.

Achondroplasia is the most common human skeletal dysplasia, occurring in ∼1 in 20 000 births worldwide.^37^ Although growth hormone therapy is not effective in improving final height in children with achondroplasia,^38^ a long-acting analog of C-type natriuretic peptide, vosoritide, provides a mechanism-based treatment for this disorder and modestly accelerates linear growth in patients with achondroplasia^39^ or hypochondroplasia^40^ by approximately 1.5 cm/year. This treatment may also reduce the risk of life-threatening stenosis of the foramen magnum, increasing interest in starting treatment in early infancy,^41^ thus requiring careful monitoring and management by the subspecialist.

#### Unnecessary Referrals

Many referrals to pediatric endocrinologists worldwide are for children who do not require treatment. In a US study, more than 40% of referrals for acquired hypothyroidism did not result in treatment, and indeed 12% of patients had referral thyroid-stimulating hormone values <6 mIU/mL,^42^ trivially above the reference range. Only 1.7% of patients referred for adolescent gynecomastia had abnormal laboratory findings.^43^ In Italy, treatment was prescribed to only 35% of referred patients.^44^

Many primary pediatric practices are set up to maximize efficiency for well-child care and for the management of acute illnesses, making it challenging for primary pediatricians to implement evaluation protocols for conditions that they encounter relatively rarely. “Mid-level” practitioners (nurse practitioners or physician assistants) are increasingly involved in primary pediatric care, especially for government-insured populations. In some circumstances, this care model can be cost-effective and does not increase referral volumes,^45^ but implementation for pediatrics in the US may be impeded owing to insufficient pipelines for pediatric nurse practitioners, necessitating utilization of family nurse practitioners who have relatively little formal training in pediatric care.^46^

Parents also influence new referrals made by primary care physicians. A survey of US pediatricians found that more than 40% sometimes or often made nominally unnecessary subspecialist referrals based on parental request.^47^ The simple fact of a referral may ameliorate parental concern; for this reason, it may be challenging to reduce referrals for parental concern, although a robust e-consult system could help.^48^

#### Referrals for Short Stature

Referrals for short stature represent a special category of problematic referrals. In Dutch studies,^49^^,^^50^ only 5%-18% of children referred for short stature had a pathological condition identified (reviewed by White et al^51^). Children referred for short stature are disproportionately White males; almost one-half of the referred children do not return for follow-up.^52^ However, parents of children referred to pediatric endocrinologists for growth evaluation, when queried 15 years later, were much more likely to report that their child had improved self-esteem, treatment from peers, and overall ability to cope with height than were unreferred parents.^53^ Growth hormone treatment is FDA approved for idiopathic short stature (albeit not well reimbursed by insurers^54^^,^^55^), often leading to parental pressure to prescribe growth hormone to normal children, a potential ethical dilemma and counseling burden.^56^^,^^57^

#### Reluctance to Appropriately Discharge from Subspecialty Care or to Comanage

Continued subspecialty care is appropriate for many pediatric endocrinology patients, particularly those with conditions (such as type 1 diabetes) requiring multidisciplinary care teams for optimal management, those treated with expensive medications that need repeated insurance authorizations (eg, growth hormone, GLP-1 agonists), or those with conditions requiring frequent medication dose titrations (eg, congenital adrenal hyperplasia). Less complex problems, such as short stature with no apparent underlying medical condition, might benefit from at most a 1-time consultation and subsequent primary care management. In principle, hypothyroidism after the first few years of life could be managed by primary care pediatricians or family practitioners, given that most hypothyroidism in adults is managed at the primary care level. However, in some cases, the pediatric endocrinologist may provide superior continuity of care, a nurse practitioner with relatively little pediatrics training may lack experience or comfort with managing endocrinologic conditions in children, the workflow in a busy primary care clinic may not afford the time to review and follow-up on laboratory results, or the lack of a common electronic medical record system may impede comanagement. Nevertheless, comanagement of appropriate conditions should be encouraged wherever possible.

### Remuneration

#### The Relative Value Unit System

The following discussion of the economics of pediatric endocrinology is specific to the US.

All physician effort in the US is denominated in relative value units (RVUs). These are the weights given by the Centers for Medicare and Medicaid Services (CMS) under the Medicare Resource-Based Relative Value System, which was implemented in 1992. There are 3 types of RVUs: (1) physician work RVUs (wRVUs), (2) practice expense RVUs, and (3) professional liability insurance RVUs. We will limit this discussion to wRVUs.

The data for developing the original wRVUs came from a 1988 study that estimated the work involved in a wide variety of patient care services.^58^ CMS is required by law to review RVU assignments at least every 5 years; it relies heavily on recommendations from the American Medical Association's Specialty Society Relative Value Scale Update Committee (the RUC).^59^

The RUC has 22 of its 32 members appointed by major national medical or surgical specialty societies and another 4 seats that rotate on a 2-year basis, with 2 reserved for an internal medicine subspecialty, 1 for a primary care representative, and 1 for any other specialty. There are several ex officio members. Although there are many surgical professional societies, surgical subspecialties are heavily overrepresented; for example, there are roughly twice as many surgeons as pediatricians in the US, but surgical subspecialties have 11 seats on the RUC, vs 1 for all of pediatrics. The underrepresentation of pediatric subspecialties on the RUC is especially striking when one considers that 21% of the US population is younger than 18 years. This has important implications for how physician effort is valued.^60^

With few exceptions, pediatric endocrinologists perform few or no procedures. We talk to our patients and their families, we examine them, we order and interpret blood tests, and we often prescribe medications. These activities are collectively referred to as “evaluation and management (E/M).” They all take time. Each encounter has a Current Procedural Terminology (CPT) code with an associate RVU value, so, for example, office visits of differing complexity of medical decision-making are expected to take different amounts of time. These might range from a minimum of 20 minutes for a routine office follow-up visit with no laboratory data being reviewed (CPT code 99213, 1.3 wRVU) to a complex new patient visit taking 60 minutes (CPT code 99205, 3.5 RVU). Looking at the time expectations for many of the visit codes used by pediatric endocrinologists, we can conclude that the RVU system values our time at 3.75 RVU per hour.

#### Reimbursement

Although reimbursement for physicians caring for older adults is largely covered by Medicare, the care of children who do not have commercial insurance (40%-60% of many clinic populations) is covered by Medicaid, which has lower reimbursement rates than Medicare (eg, $33/RVU for Medicare vs $28/RVU for Texas Medicaid). At 3.75 RVU per hour, Medicaid gross reimbursement would amount to $105 per hour, which would not support competitive salaries, even before deductions for overhead or institutional taxes, and even assuming 40 hours per week in clinic with no vacation time.

#### Support for Graduate Medical Education

Graduate medical education (residency and fellowship training) in fields outside of pediatrics is supported by approximately $17 billion in annual payments from the CMS through Medicare. Children's hospitals do not receive such payments; instead, 59 hospitals share approximately $369 million in payments from the Children's Hospitals Graduate Medical Education program in 1999 under the Health Resources and Services Administration—approximately 2% as much,^61^^,^^62^ but the 3185 pediatrics residents who matched in 2026 comprise approximately 7% of the total 44 344 positions in the match.^63^ This marked disparity in funding starves children's hospital for resources, contributing to low salaries for hospital-based pediatricians and pediatric subspecialists.

### Documentation Burden

The alternative to time-based billing is billing on complexity. The criteria for evaluating complexity have evolved over time, but the system in place for many years was arcane, burdensome in which to document, and stressful to clinicians.

#### Implementation of Electronic Health Records

As all clinicians know, the implementation of electronic health records (EHRs) has transformed medical care and led to tremendous improvements in reliability, portability, legibility, and potentially, automation. However, expectations for detailed documentation have correspondingly increased, leading to significant time demands on physicians in “conceptual” subspecialties such as endocrinology, rheumatology, and infectious diseases.

Very large studies of users of the 2 most common EHR systems from Cerner (Cerner Millennium, ie, Oracle Health) and Epic (Epic Systems Corporation), published in 2020 and 2024, respectively, yielded very similar results. Pediatric endocrinologists spent an average of almost 20 minutes per outpatient encounter in the Cerner EHR, one of the longest times of any pediatric subspecialty, exceeded only by infectious disease and rheumatology. Approximately 12% of this time was after hours. In a hypothetical workday of 18 encounters, a pediatric endocrinologist spent 6 hours in the EHR.^64^

Similarly, pediatricians spent 6.4 hours in the Epic system per 8-hour clinic day, but ∼35% of this time was spent after hours or on unscheduled days. For endocrinologists, the total was 7.7 hours spent in the EHR per 8-hour clinic day.^65^

The drivers for increased documentation come from the nature of reimbursement for E/M services, wherein more RVUs are awarded for higher levels of service. Payors require accurate documentation that supports the billed level of service. Historically, accuracy has been enforced by the US federal government.

The 1995 CMS Documentation Guidelines for Evaluation and Management Services, revised slightly in 1997, established a framework for documenting and coding patient visits based on a combination of history, examination, and medical decision-making. Key components of documentation included the reason for the visit, relevant history, physical examination findings, assessment/diagnosis, and the plan for care. At the same time, the Office of the Inspector General of the US Department of Health and Human Services, in cooperation with the US Department of Justice, instituted a nationwide initiative, eventually termed as Physicians at Teaching Hospitals audits, to review teaching physician compliance with Medicare billing rules. Several medical schools negotiated settlements totaling $68 million for overbilling Medicare. Subsequently, medical schools were given the option of conducting self-audits at their own expense to ensure regulatory compliance. Areas of interest included whether teaching physicians who billed Medicare for services furnished by residents provided sufficient “personal direction” in the delivery of the service. That question will not be discussed here. The second area of attention was whether teaching physicians were “upcoding,” that is, billing using a code level higher than the level of service that was actually performed. Level-of-service determinations were made by internal medical reviewers. The reviewers could communicate audit findings to clinicians and could mandate more intensive scrutiny of clinicians who “upcoded” more often.

The internal audit system, although necessary in the compliance situation at the time, was stressful for pediatric endocrinologists because of perceived pressure to overdocument to avoid accusations of “upcoding.”

It was eventually generally recognized that this system was extraordinarily burdensome in which to document, and in 2021, it was extensively revised by CMS. The history and physical examination were eliminated as determinants of the code level, and the focus shifted to the alternatives of documenting either medical decision-making (the number and complexity of problems addressed, the amount of data reviewed, and the risk of complications and/or morbidity or mortality) or, instead, total time spent. Time included both face-to-face and non-face-to-face time spent by the practitioner on the date of the encounter. A similar revision was put in place for inpatient encounters in 2023. The effects of these changes on the documentation burden have not yet been extensively studied.

## Fellowship Training

What is the optimal length of fellowship training?

### Goals of Training

Decisions as to the necessary length of training depend on the goals of that training and methods of evaluation. In the case of a pediatric subspecialty, questions regarding the length of training pertain to both general pediatric training and the subspecialty. The required length of general pediatrics residencies increased from 2 to 3 years in 1974 and has remained stable since. Although pediatric endocrinology as a field has existed since 1935 as previously discussed, the Endocrinology subboard of the ABP was not established until 1978, and it was possible to be admitted to take the certifying examination without a formal fellowship in pediatric endocrinology until 1992. The 3-year duration of modern pediatric subspecialty fellowships was adopted to provide pediatric endocrinologists with training in both clinical medicine and medical research, which was consistent with the early development of the field within academic medical centers. It is questionable whether this is an appropriate basic requirement under current circumstances, in which there is relentless pressure from administrators for clinicians to spend almost all (in many institutions, 90%-95%) of their time in clinical activities. It is noteworthy that adult endocrinology fellowships remain at 2 years, and as noted previously in this review, the match fill rate for adult endocrinology fellowships is >98% vs 60% for pediatric endocrinology fellowships.

Several arguments can be made for 3-year fellowship training. One is that medical knowledge has expanded rapidly, increasing the amount of subject material that must be mastered. As one crude measure, a standard text, *Nelson Textbook of Pediatrics*, increased in size from 2170 pages in 1979 to 4264 pages in 2020. Another is that duty hours limitations, which were instituted by the Accreditation Council for Graduate Medical Education (ACGME) in 2003, reduced unsafe working conditions for residents but necessarily reduced clinical experience.^66^^,^^67^ Moreover, increased requirements for in-person supervision by attending physicians to bill (as discussed previously) mean that fellows never manage even simple patients without supervision until after they graduate.

### Evaluation Methods

Other than board certification and in-training examinations administered by the ABP, there was no mandated formal framework for evaluating fellows until 1999, when a framework of Core Competencies was adopted by the ACGME. These remain in widespread use throughout medicine (and in other professions such as nursing) and are included on many hospital credentialing evaluation forms. They are currently evaluated using an assessment tool, the Milestones, which was introduced by ACGME in 2013 with a revised and simplified version 2.0 introduced starting in 2016. This evaluation system of “competency-based medical education” has been reformulated in recent years as “entrustable professional activities (EPAs). Each EPA includes a particular combination of different Milestones. The ABP plans to incorporate EPAs into its certification decisions, with a target of 2028.^68^^,^^69^

Several 2025 studies^70^, ^71^, ^72^ suggested that many fellows were not ready for independent practice after 2 years, based on EPA scoring frameworks. Moreover, almost 10% of pediatric fellows did not meet the expected supervision level for clinical EPAs at graduation, yet more than 70% of these apparently unready fellows passed their subspecialty certification examination.^70^ The significance of this is uncertain. Although it is obvious that medical knowledge (as measured by examination performance) is only one component of actual competency, at least it can be measured objectively. In contrast, most competency-based evaluation tools are inherently subjective, many medical faculty may reverse-engineer their scores based on “gut impressions,”^73^ and faculty may automatically rate fellows more highly as training progresses because that is the expectation. Many of the Milestones, in their emphasis on advocacy, quality improvement, and efficient transition of care, seem aspirational rather than reflecting the reality of practice environments in which few pediatric endocrinologists spend much time on these activities.

Regarding the research requirement, only 14% of full-time pediatric endocrinologists spend >25% of their time in such endeavors and only 6% spend >50% of their time in research, as previously noted. Only 1% of pediatric endocrinologists report being employed in industry.^74^ However, 13% of fellows hope to have research careers, suggesting a need for more effective career counseling in many fellowship programs.^75^ Few practice environments at medical schools or hospitals can support unfunded research. Although industry-sponsored clinical research may be a worthwhile activity in many cases, it generally doesn't contribute much to academic promotion decisions, which expect investigator-initiated research accomplishments. But it has been challenging to obtain funding to support investigator-initiated research in recent decades; for example, in the past decade, no more than 16% of “R01” research grant applications have been funded by the National Institute of Diabetes, Digestive and Kidney Diseases, one of the main grantors for endocrinology research.^76^ Moreover, recent (mid-2026) changes in National Institutes of Health funding proposed by the federal Office of Management and Budget may compromise peer review and substantially increase uncertainty in funding.^77^

## How to Proceed

“And you may ask yourself, ‘How do I work this?’”

Talking Heads, Once in a Lifetime.^1^

Many of the issues we've discussed might best be addressed in the context of comprehensive health care reform in the US^78^ but that has been historically difficult to implement and is beyond the scope of this review. In summary, a solution to workforce challenges must include addressing financial barriers through improved compensation and reimbursement reform that reflects the time-intensive nature of care. Recognition of nonclinical responsibilities is also essential.

### Value-Based Compensation

As previously noted, the current RVU-based compensation system is set up to reward procedures. It doesn't acknowledge or remunerate activities such as program development, quality improvement, teaching, or scholarship, leading to negative incentives for pediatric endocrinologists to participate in any of these activities.^79^ Moreover, if on-call availability is not remunerated, as is often the case, this creates pressure to work additional clinical sessions to make RVU quotas, leading to sleep deprivation, fatigue, and burnout.^80^ Accordingly, some have advocated for value-based compensation that does reward these activities.^8^^,^^79^

Although the incentives for hospitals to ensure adequate staffing for inpatient activities are obvious, there are also strong incentives to support outpatient clinics. These include minimizing hospitalizations of at-risk patients (such as those with type 1 diabetes) by providing timely preventive and follow-up outpatient care. Furthermore, these activities generate downstream income from laboratory tests, dynamic endocrine testing (such as growth hormone or cosyntropin stimulation tests), radiologic imaging (including bone age studies, dual-energy X-ray absorptiometry scans, and magnetic resonance imaging), and infusion services for stage 2 and stage 3 type 1 diabetes (teplizumab) and metabolic bone disease (zoledronic acid).^8^ In particular, the ability of hospital pharmacies to “buy and bill” for infusible drugs^81^ represents a potential income stream as previously noted.

It is essential that pediatric endocrinologists’ salaries become competitive with other pediatric subspecialties. If hospitals do not employ physicians directly, a robust multiyear “funds flow” (or “gains-sharing”) agreement between each hospital and medical school can allow stable budgeting given that it is not possible to completely cover pediatric endocrinology salaries from collections, particularly in markets with substantial patient populations with government insurance.^82^, ^83^, ^84^, ^85^

Although there has been a recent surge in unionization efforts by physicians to improve compensation and working conditions,^86^ the practical and legal obstacles to such a course of action are beyond the scope of this review.

### Length of Training

Notwithstanding questions about fellow competencies arising from EPA evaluations, the system must change to reduce the opportunity cost of pursuing a pediatric endocrinology fellowship. We strongly advocate for reducing formal training by 1 year, at least for fellows planning clinically oriented careers. This would be consistent with current requirements for adult endocrinology training and reflects the reality that few pediatric endocrinologists spend much time on research. Refinements to the system could promote readiness to practice. Pediatrics residency and fellowship could be better integrated; the precedent is pediatric neurology, which requires a 5-year residency after medical school, including 2 years of general pediatrics and 3 years of child neurology, with one of those years including adult neurology training. Additionally, it should be kept in mind that very few pediatric endocrinologists enter solo private practice upon graduating fellowship; instead, they are likely to enter medical school or hospital-based group practices, and these environments can provide robust opportunities for mentoring and supervision. Moreover, US hospitals mandate Focused Professional Practice Evaluations for all new privileges granted to staff physicians, which represents a potential enforcement mechanism to assure the competence of new fellowship program graduates. In principle, these could be coordinated with the ABP to assure that competence in all relevant EPAs has been properly documented. The idea of 2-year, competency-based fellowship training has very recently been endorsed by the ABP.^87^

### Reorganization of Care Delivery

As noted previously, many referred patients could, in principle, be managed by primary care physicians.^12^ Screening protocols implemented by primary care physicians should be able to reduce the volume of such referrals, but this requires an effective and efficient system to communicate recommendations for workup (including recommendations for no workup) to the referring physician in a way that does not require nonremunerated time by the pediatric endocrinologist. This could best be accomplished with an electronic referral portal,^88^ perhaps eventually incorporating artificial intelligence (AI) triage assistance supervised by appropriately trained nursing personnel. Additionally, a system for e-consultation must be developed that compensates the endocrinologist for the time required to review the results and communicate the evaluation to the referring physician.^48^

Other innovations could reduce practice burdens. A nurse line working with established protocols can effectively answer 70% of after-hours calls from diabetes patients’ families in the community, dramatically reducing the number of calls that must be answered by the on-call physician.^89^ Moreover, when the family of a sick patient calls the nurse line first, the patient is likely to be considerably less ill on arrival in the emergency department and more likely to be discharged rather than admitted.

We have deliberately not discussed the role of AI because of the very rapid pace of its development, but it is already apparent that documentation burden can be at least slightly reduced with the use of ambient AI “scribes” to streamline the documentation of outpatient visits.^90^^,^^91^

## Conclusion

Although many find pediatric endocrinology a fulfilling career, workforce shortages lead to increased workloads, and relatively low remuneration and excessively long training requirements discourage entry into the field. This situation risks adverse effects on child health, particularly in geographic areas with relative scarcity of pediatric endocrinologists. Potential solutions include value-based remuneration supported by robust funds flow agreements with hospitals, streamlined training requirements, and utilization of informatics to support more targeted referrals and effective comanagement of selected patients with primary care physicians.

## Declaration of Competing Interest

The authors declare that they have no known competing financial interests or personal relationships that could have appeared to influence the work reported in this paper.
